# Covering digital health applications in the public insurance system: how to foster innovation in patient care while mitigating financial risks—evidence from Germany

**DOI:** 10.3389/fdgth.2023.1217479

**Published:** 2023-10-11

**Authors:** Nicole Groene, Luca Schneck

**Affiliations:** Department for Health and Social Sciences, FOM University of Applied Sciences, Munich, Germany

**Keywords:** digital health applications, digital therapeutics, reimbursement, statutory health insurance, resource allocation, healthcare innovation

## Abstract

**Context:**

Digital health applications that support patients in managing their condition can have a positive impact on patients' health and improve the overall care process. In late 2019, as the first country worldwide, Germany included digital health applications in the benefit basket of the statutory health insurance (SHI) system to enable fast, broad-scale patient access and encourage innovation in the digital health industry. While the policy is widely recognized as a pioneering step toward improving patient care through digital technologies, there are concerns regarding the mechanics of the policy and the resulting financial risks for the SHI system.

**Goals:**

The primary objective of this article is to provide a comprehensive and balanced overview of the German policy by evaluating its success in achieving its goals and by reviewing challenges that have emerged. The secondary objective is to delineate prospective policy options and areas warranting future research.

**Approach:**

The article analyzes publicly available data of the Federal Institute for Drugs and Medical Devices collected between February 1st and July 17th, 2023, and complements it with empirical findings published by academic institutions and sickness funds. It discusses policy options and related areas of future research to overcome the identified challenges without jeopardizing the purpose of the legislation to encourage innovation in the digital health industry to improve patient care.

**Conclusion:**

In line with the goals of the reimbursement policy, the inclusion of digital health applications in the SHI benefit basked has entailed new digital treatment options for patients across multiple disease areas. However, from a health policy perspective, the policy has several shortcomings, including low prescription rates, the temporary reimbursement of digital health applications that lack proven benefit, and a pricing framework that does not take into account the efficacy and efficiency of a treatment and may lead to a suboptimal allocation of public resources. Rather than the public system covering digital health applications without proven benefit, the authors suggest giving SHI organizations more budget authority to directly incentivize research and development activities and to introduce value-based pricing. More research is needed to determine the details of these mechanisms.

## 1. Introduction

Digital health applications (DiHAs) are interactive mobile or desktop applications that provide treatment or support treatment for diseases (1). These digital therapeutics aim to improve care and population health within a wide range of disease areas such as mental health, cardiovascular, and musculoskeletal conditions (2). Many DiHAs support patients in implementing lifestyle changes or guide them through behavioral therapy or exercise programs for musculoskeletal conditions or mental health problems (3). As such, they empower patients to improve their health and well-being while also offering the potential to bridge the capacity constraints inherent in traditional patient care. For example, in Germany, patients must wait 142 days from their first contact with a psychotherapist to begin psychotherapy (4). During this waiting period, symptoms may be alleviated via app-based support.

With approximately 74 million insured individuals, Germany has one of the largest statutory health insurance (SHI) systems in the world (5). In late 2019, the Parliament passed the Law for Better Care through Digitalization and Innovation, also referred to as the Digital Healthcare Act (Digitale-Versorgung-Gesetz) (6). The legislative process was accompanied by the “I.DiGA project” funded by the Ministry of Health which involved experts from academia as well as the private sector, including entrepreneurs and managers active in the digital health industry (7). Using an iterative and agile methodology, the project developed approaches for the categorization, evaluation, pricing and reimbursement of DiHAs and its results informed the legislation (2). The adopted innovation-friendly law was considered a pioneering step toward digitally-supported patient care both nationally and internationally (8). Its main purpose was to provide patients with broad access to low-risk digital health applications and drive innovation in the domain. The rationale regarding the latter is that if there is the prospect of reimbursement, more digital health applications will be developed by researchers, start-ups, or established companies—a view that has also been emphasized by the digital health industry (9) and is in line with findings that uncertainty about funding is an important barrier for innovation in healthcare (10) and that small and medium enterprises (SMEs) in particular struggle to meet the requirements of evidence generation sought by public payors (11).

The law entitles all individuals covered by SHI, approximately 90% of the population, to use DiHAs (12, 13). As a consequence, all independent SHI funds are required to cover the cost of a digital health application if (i) the application has been approved by the German Federal Institute for Drugs and Medical Devices (Bundesamt für Arzneimittel und Medizinprodukte, BfArM) and (ii) the patient is eligible for the DiHA, which primarily requires that the patient is diagnosed with the condition the DiHA has been approved for (14). To further encourage and facilitate innovation in the domain, the regulator has set low hurdles for the approval process. This relates to the required level of evidence, the ease and speed of the assessment process, and the rules governing the pricing (8).

While this legislation has been widely recognized as forward-looking and a driver of digital health innovation (12), it also entails risks for the public healthcare system, especially those that are financial (15); concern has been voiced in particular by individual SHI funds and the Federal Association of Statutory Health Insurance Funds (GKV-Spitzenverband, GKV-SV) (16). Countries seeking to introduce similar innovation-friendly legislation might therefore refer to Germany to understand the mechanics of the legislation, its related risks and how these risks have empirically unfolded to derive implications for their national policies.

Against this background, this article describes the coverage policy of DiHAs in Germany and how it was set up to encourage digital health innovation. Section 3 introduces and applies a framework to evaluate the initial success and challenges that have emerged. Success is defined as to whether the policy has achieved its goals of fostering innovation in digital health and enabling broad-scale access to DiHAs. Challenges relate to financial risks for the public healthcare system that result from the coverage policy. Section 4 discusses policy options that alleviate the shortcomings of existing legislation. The article concludes with a summary of areas of future research and recommendations on how to further develop the existing reimbursement policy to achieve its goals more effectively and with less financial risk.

## 2. Reimbursement policy of digital health applications in Germany

Under the Digital Healthcare Act, patients in Germany who fall under the SHI system are entitled to coverage benefits regarding DiHAs. Such DiHAs are also referred to as “prescription apps” and are listed in an official online directory (3). A DiHA is considered a lower-risk medical device classified a class I or class IIa medical device under EU Medical Device Directive 93/42/EEC [formerly EU Medical Device Regulation 2017/745] (17, 18). Thus, all DiHA must carry a CE mark which indicates that the medical product complies with European regulations for safety, health and environmental protection (19).

### 2.1. DiHA approval process and requirements

To receive the status of a DiHA and thus be included in the benefit based of the SHI, health applications must undergo an approval process by the BfArM. To set low hurdles for innovators and in line with the Digital Health Applications Regulation (Digitale Gesundheitsanwendungen-Verordnung, DiGAV) (20), the BfArM introduced a so-called “Fast-Track Procedure” (21), a three-month process during which the authority checks the compliance of an app with requirements regarding safety, functional capabilities, data security and protection, and general quality. Additionally, to be listed in the directory of approved DiHAs, app manufacturers must provide evidence of “positive healthcare effects”. These effects may be medical benefits or “patient-relevant improvements of structures and processes” (“patientenrelevante Struktur- und Verfahrens­verbesserungen”, pSVV). PSVV designation requires the app to provide substantial support for patient activities such as adherence, coping with illness in everyday life, or coordinating various treatment procedures. If manufacturers cannot present studies proving a positive healthcare effect upon application for approval, they may still obtain the status of “provisional listing” if they can plausibly demonstrate that a planned trial will generate sufficient evidence. After 12 months of provisional listing, DiHA manufacturers must present bespoke studies to obtain the status of “permanent listing.” If this is not possible, manufacturers can request an extension of this provisional period to 12 months. After this extension period, a DiHA will either be granted the status of “permanent listing” or be delisted from the DiHA directory. Manufacturers can request the removal of their DiHAs from the directory at any time.

### 2.2. Patient access to DiHAs

The two routes by which patients with SHI coverage in Germany can access DiHAs include a prescription by a physician or licensed psychotherapist, currently representing 89% of all instances (16), or direct patient application to their SHI fund that will be approved if patients provide proof of the respective diagnosis, a practice often referred to as “self-prescription”. The patient must meet the criteria that the DiHA has been approved for, such as age, gender, and lack of contraindications. Currently, the rate of self-prescribed DiHAs varies greatly, from 5% for musculoskeletal conditions to 39% for urogenital conditions, including impotence and endometriosis (16). In either case, patients must request an activation code from their SHI fund to use a DiHA free of charge and the SHI fund must reimburse the manufacturer once a prescribed DiHA has been activated by a patient.

### 2.3. Pricing of DiHAs

SHI funds must reimburse manufacturers for each prescribed or self-prescribed DiHA activated by patients. The cost of 90-day access to a DiHA currently ranges from EUR 119 for Mawendo (Mawendo GmbH, Germany) for patients with knee disease to EUR 2,077.40 for levidex (GAIA AG, Germany) for patients with multiple sclerosis (MS) (3). This wide range may be attributable to the fact that during the 12 months following the initial listing, manufacturers are free to set prices with only a few boundaries. Only thereafter will the GKV-SV negotiate prices with manufacturers. If no agreement can be reached during negotiations, an arbitration court (22) will set the price.

**Figure 1:**
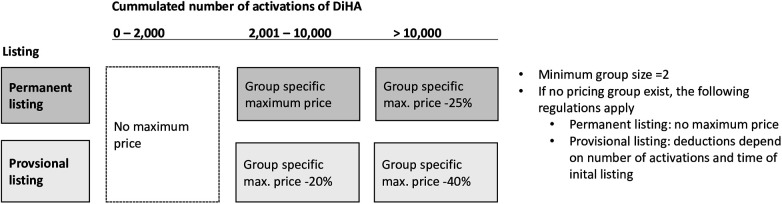
Price ceilings for DiHAs depending on status and number of activations.

Owing to strong contentions regarding the cost of DiHAs and the trend towards higher prices set by manufacturers, price ceilings were introduced in October 2022 for the four most common groups of DiHAs. These ceilings were based on freely-set prices of existing DiHAs. While manufacturers and the GKV-SV have yet to negotiate reimbursement prices that apply after month 12 of listing; these maximum prices constitute an upper limit to what may be quoted and negotiated. The current 90-day cost limits are for musculoskeletal (EUR 321.30), neurological (EUR 802.80), mental health (EUR 599.40), and endocrine (EUR 513.90) conditions. In April 2023, three additional groups were added: otolaryngology (EUR 225.90), cancer (EUR 1,049.40), and urogenital conditions (EUR 670.50) (23). As Figure 1 shows, these price ceilings apply only after a DiHA has been activated more than 2,000 times. For DiHAs with permanent listings that have been activated more than 10,000 times, the price ceiling is reduced by 25%. For provisional DiHAs with more than 2,000 and up to 10,000 activations, these price ceilings were reduced by 20%, and for provisional DiHAs with over 10,000 activations, ceilings were reduced by 40%. If no group of comparable DiHAs exists, the manufacturer may set the initial price freely.

The above-described approval process and reimbursement rules of DiHAs differ in part substantially from other SHI service areas such as pharmaceuticals and thus entail more risks for public sickness funds.

• Provisional approval: In contrast to the pharmaceutical sector in which manufacturers need to provide evidence of drug efficacy to obtain approval and reimbursement (24), SHI funds are obliged to reimburse DiHAs with provisional approval status, i.e., when there is no scientific proof yet that the product entails a patient benefit.
• Level of scientific evidence: Evidence requirements for DiHAs to obtain permanent approval and to determine reimbursement prices are also lower than for other areas. For pharmaceuticals, the Federal Joint Committee (Gemeinsamer Bundesausschuss, G-BA) mandates rigorous scientific standards on how to determine the therapeutic benefit of a treatment (25). The G-BA regularly commissioned the Institute for Quality and Efficiency in Health Care (Institut für Qualität und Wirtschaftlichkeit im Gesundheitswesen, IQWiG) with the assessment of drugs. In accordance with the rules of the G-BA procedure, the IQWiG considers randomized controlled trials (RTCs) and only uses nonrandomized interventions or observational studies in exceptional, justified cases (26, 27). DiHA manufacturers, in contrast, may provide evidence of patient benefit through retrospective comparative studies or retrospective intraindividual studies (28). Therefore, some of these studies have been shown to contain potential biases (28, 29).
• Patient-relevant improvements of structures and processes: Compared to pharmaceuticals, the regulator has substantially expanded the definition of patient benefit of DiHAs. For pharmacological treatments, the efficacy is to be measured as patient-relevant outcomes in terms of mortality, morbidity, and health-related quality of life related to an appropriate comparator (26). In contrast, DiHAs may not only be permanently approved and reimbursed for health-related outcomes but also for entailing patient-relevant improvements of structures and processes.
• Pricing process: The pricing mechanism for pharmaceuticals and DiHAs show similarities, in particular the negotiation of reimbursement prices between manufacturers and the GKV-SV within 12 months of approval. For pharmaceuticals, such negotiations take place if the drug offers an added therapeutic benefit vs. an established drug, otherwise reference prices apply (30). For DiHAs, there is no formal approach on how to quantify the benefit of a DiHA relative to a comparable treatment to inform price negotiations. Instead, the abovementioned group-specific price ceilings apply.

Overall, in an internationally pioneering step, the German regulator has created a new SHI service area with a facilitated and fast approval process (21) as well as lower or limited evidence requirements to foster digital innovation and enable fast, broad-scale patient access to it.

## 3. Assessment of policy and need for refinement

This section presents an initial comprehensive assessment of the DiHA reimbursement policy approximately 3.5 years after its inception from the point of view of the public regulator. With regard to health, it is the responsibility of the regulator to ensure medical and custodial care of the population through adequate policies; this includes enabling access to healthcare and fostering the efficiency of services delivered within the healthcare system (31). The government and its subordinate agencies aim to protect and improve the health of the population (32, 33). In line with this, the goal of the German DiHA reimbursement policy and the Fast-Track-Procedure is to foster the availability of new digital treatment options and to enable fast, broad-scale access to them (34).

**Figure 2:**
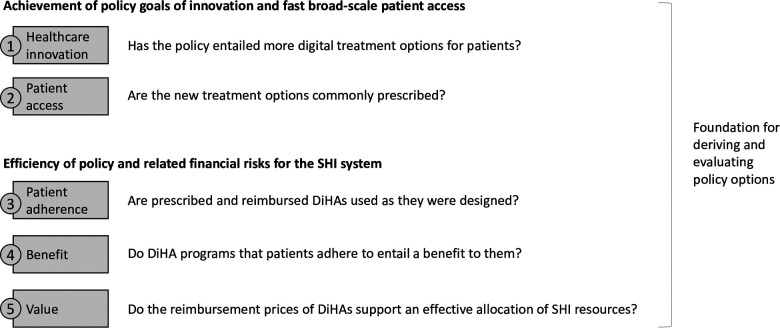
Criteria to assess the efficiency of the German DiHA coverage policy and related challenges for the public healthcare system.

Applying the framework displayed in Figure 2, we first evaluate if and to what extent the desired effect of the policy has materialized, namely:

(1) Healthcare innovation: Has the policy entailed more digital treatment options for patients?
(2) Patient access: Are these new treatment options commonly prescribed?

Second, we evaluate financial challenges for the SHI system related to the reimbursement policy. In line with the principle of cost-efficiency, services and benefits covered by the SHI system must be “adequate, appropriate and efficient” and may “not exceed the measure of what is necessary” according to paragraph 12 and similarly paragraph 70 of The Fifth German Social Code Book (SGB V) (35). Although measuring cost efficacy in healthcare is a topic of continuous political, legal, ethical, and academic debate in Germany, there is wide agreement that public health insurance funds should not be allocated to unnecessary or ineffective treatments (36, 37) and that drugs and medical devices should not be excessively priced (38). Thus, to assess the efficiency of the legislation from a resource perspective, we investigate the following questions:

(3) Patient adherence: Are prescribed and reimbursed DiHAs used as they were designed?
(4) Benefit: Do DiHA programs that patients adhere to entail a benefit for them?
(5) Value: Do the reimbursement prices of DiHAs support an effective allocation of SHI resources?

According to our evaluation framework, to have a clear and sustainable positive impact on the health of the population, these questions should be answered “yes”. In the absence of quantitative targets of the policy, it is not possible to size the success of the policy through this approach. It is, however, possible to obtain a directional answer, to systematically identify shortcomings of the regulation and to derive options on how to improve the policy to achieve its goals more effectively.

To investigate those five aspects, we collected publicly available data from the BfArM on DiHAs that have been approved since the inception of the law (3). We started the data collection process in February 2023. The last time we updated the collected dataset was on July 17th, 2023. Thus, all numbers reported on the status of DiHAs in the German market are based on the status quo in mid-July 2023. We further complemented this information with data reported by the GKV-SV reflecting the status quo of DiHA usage in the SHI system as of September 30th, 2022. Wherever these data were not suitable to answer our policy assessment questions, we reviewed the published literature to identify relevant studies investigating this aspect.

In the subsequent paragraphs, we apply our assessment framework as follows: For each aspect and the related policy assessment question, we briefly explain its rationale, present analyses on the status quo—either from our primary data collection effort or from published studies—and draw preliminary conclusions that will be synthesized in the policy options section. Wherever suitable to support our preliminary conclusions, we complement the section with information on the current political and academic debate.

### 3.1. Healthcare innovation and availability of digital therapy options

#### 3.1.1. Rationale

For the DiHA policy to increase medical care options for the population, manufacturers must develop digital therapeutics, obtain a CE mark and undergo the approval process by BfArM. Going through this process—even for apps that had been developed already but are lacking the required certification and approval—is time intensive and requires skilled and well-trained staff that many start-ups and SMEs lack of (11). Approval and reimbursement processes may thus constitute barriers to innovation in digital health (10, 11). Thus, when evaluating the DiHA policy, the first aspect to be investigated is if it has entailed new digital therapy options available to the SHI population, i.e., if manufacturers have successfully undergone the process.

#### 3.1.2. Status-quo

The data collected from the official DiHA directory show that since the inception of the law, 54 DiHAs have been approved covering over 10 disease areas and 30 indications. Six of them have been delisted thus far leaving 48 provisionally or permanently approved DiHAs as of mid-July 2023. As shown in Table 1, the most common conditions addressed by DiHAs are mental health conditions with a total of 22 DiHAs available. Within this disease area, almost one third of the DiHAs target patients suffering from mild to moderate depression, which is mostly treated via digital cognitive behavioral therapy in line with the latest treatment guidelines for mild unipolar depression (39). Musculoskeletal (5 DiHAs), neurology (5 DiHAs) and endocrine including obesity (4 DiHAs) are further disease areas where there are several digital treatment options now available to patients. For other common disease areas such as gastrointestinal, otolaryngology and respiratory diseases, the number of available DiHAs is limited to one or two, some of which target highly specific conditions such as tinnitus. No DiHA exists for example for rare diseases, pediatrics or infectious diseases.

**Table 1:** Overview of the number of listed DiHA by disease area [number of DiHAs in directory /of this number of DiHAs with provisional listing as of July 17th, 2023].

Disease area	Indications	Main therapeutic approaches
Mental health [22/10]	Alcoholism [1/0], anxiety [5/1], binge eating [1/1], borderline [1/1], bulimia [1/1], depression [7/4], insomnia [2/1], panic [1/0], stress and burnout [1/0], tobacco addiction [2/1]	Cognitive behavioral therapy (CBT) [20/8], dynamic dialog and exercises [1/1], psychotherapy [1/1]
Musculoskeletal [5/3]	Back pain [1/0], back, knee and hip pain [1/0], disease of the patella [1/1], kneecap disease [1/1], osteoarthritis of the knee [1/1]	Physiotherapy [4/2], exercise therapy [1/1]
Neurology [5/4]	Aphasia [1/1], migraine [1/1], mild neurocognitive disorder [1/1], MS fatigue [1/0], MS [1/1]	Standard speech therapy [1/1], seizure prophylaxis, nutrition reports, headache diary [1/1], CBT [2/1], multidomain cognitive training [1/1]
Endocrine [4/2]	Diabetes type 2 [2/2], obesity [2/0]	Diabetes control and self-management [2/2], multimodal obesity therapy [2/0]
Oncology [2/2]	Breast cancer [2/2]	CBT [1/1], support and coping strategies [1/1]
Gastrointestinal [1/1]	Irritable bowel syndrome [1/1]	CBT and diet support [1/1]
Otolaryngology [2/1]	Tinnitus [2/1]	CBT [1/0], education and self-management [1/1]
Urogenital [2/1]	Endometriosis [1/1], impotence [1/0]	Multimodal therapy and symptom documentation [1/1], exercises, mindfulness, endurance, and sexual therapy [1/0]
Respiratory [1/1]	COPD [1/1]	Movement, respiratory exercises, education [1/1]
Cardiovascular [1/1]	Heart failure [1/1]	Self-management, incl. monitoring and education [1/1]
Other [3/1]	Chronic pain [2/1], vaginismus [1/0]	CBT [3/1]

#### 3.1.3. Preliminary conclusion

While these data do not prove a causal relationship between the legislation and the level of innovation in the industry, they indicate that the regulation has entailed more digital therapy options in Germany. With a total of 48 approved DiHAs in July 2023, the introduction of the Digital Healthcare Act led to an increase in digital treatment options that are now available to patients in the SHI system (40). Thus, the policy can be deemed effective in this regard.

Prior to the Digital Healthcare Act, digital health applications were only available to patients in the private pay market or under specific circumstances, typically in case there was a dedicated selective agreement (“Selektivvertrag”) between one SHI fund and a manufacturer to cover the cost of a digital solution (9). With approximately 200 different SHI funds in Germany, only a small fraction of the population was covered by a selective agreement. Otherwise, for an innovative digital healthcare product to be included in the benefit basket of the SHI, it took approximately five years (41). Thus, access to digital health applications was substantially more limited than it is currently.

Prior to the Digital Healthcare Act, there were also fewer incentives for the private sector to develop digital therapeutics. For manufacturers and potential innovators, negotiating selective agreements with multiple SHI funds constitutes a substantial effort. Furthermore, the uncertainty of whether a manufacturer would later be able to negotiate reimbursement contracts with SHI funds created a reluctance to invest in research and development in digital therapeutics, often limiting financing options to social investors rather than accessing the entire venture capital landscape (42). While there is not sufficient data to prove that the DiHA regulation has led to more research and development (R&D) activities, the prospect of reimbursement of effective health apps is likely to have reduced barriers to innovation.

Another indicator of the initial success of the policy in fostering the availability of digital health innovation for patients within the SHI system is that other European countries have been actively studying German legislation for its transferability (40). Belgium was the first country to follow the German example and introduce a similar policy in 2021 (43). With the Social Security Financing Act for 2023 (Loi n° 2022-1616 du 23 décembre 2022 de financement de la sécurité sociale pour 2023, LFSS) (44) France also enabled the reimbursement of health apps via the SHI system. Under the early access to reimbursement for digital devices directive (Prise en charge anticipée numérique, PECAN) the French regulator allows manufacturers to be reimbursed for one year while the proof of clinical or organization benefit has not yet been finalized (45). Recently, Austria announced its plans to allow the prescription of health applications (46).

### 3.2. Access and prescription intensity

#### 3.2.1. Rationale

To improve the health of the population, DiHAs must not only be available in the market. Patients must also obtain access to them through a prescription by a physician or a therapist, or via the route of “self-prescription”. Therefore, (self-)prescription intensity is an important criterion for evaluating the initial efficacy of the German reimbursement policy for DiHAs.

#### 3.2.2. Status quo

According to numbers published by the GKV-SV, until September 2022, 203 thousand DiHAs were prescribed or self-prescribed (16). With approximately 4,500 DiHAs prescribed in March 2021, this constitutes a cumulative average monthly growth rate of 5.6%, which is similar to the average monthly growth rate of available approved DiHAs of 5.8%. Of those DiHAs that were prescribed until September 2022 (164 thousand DiHAs), 81% were also activated during the reporting period. Thus, on average, throughout Germany, DiHAs were activated between 40 times and once per day (16).

These numbers are low relative to the size of the German SHI population of approximately 74 million and the prevalence of the conditions in the adult population that DiHAs address. For example, approximately 20% of the adult population suffers from 3-month back pain, approximately 7.2% from diabetes and approximately 19% from adiposities (47, 48). In contrast, by September 2022, the two approved DiHAs for adiposities [Zanadio (AidHere, Germany) or Oviva Direct (Oviva AG, Switzerland)] had only been activated 30 thousand times. This indicates that the prescription rate is below 0.5% for this condition.

#### 3.2.3. Preliminary conclusions

Even though new digital therapy options are available on the market, in practice, patients do not have access to them on a broad scale. Digital therapeutics constitute scientifically proven, noninvasive, and nonpharmacological treatment options (49). However, as they are currently not commonly prescribed, the policy has not yet achieved its full potential in improving the health of the population. The GKV-SV notes that DiHAs are not yet part of routine care (50). An investigation of the root causes of this low uptake is instrumental in deriving policy options.

According to the GKV-SV, 89% of activated DiHAs were prescribed by physicians, and the remaining 11% were self-prescribed (16). Although physicians are the most important stakeholders influencing patients' access to DiHAs, studies have revealed that they are slow to adopt and prescribe digital therapeutics (51, 52). The main reasons for this were limited knowledge and information, insufficient reimbursement and insufficient medical evidence, which will be discussed in more detail in the subsequent sections. Prescribing DiHAs is time-consuming, as physicians must familiarize themselves with therapeutic concepts and interventions to explain the program to patients, which may also include addressing their skepticism toward technology and resistance to behavioral changes. Furthermore, there is a general lack of knowledge and experience regarding the use of digital technology for therapeutic purposes. While there are physicians—especially those organized in digitalization-oriented physician associations—who see DiHAs as a reliable tool to improve the relationship with their patients and improve therapy compliance, mobility, and patient education (53), in a survey conducted in 2020, 70% of general practitioners (GPs) in Germany reported having no experience with healthcare apps for themselves or their patients (54). Only two-thirds of respondents were willing to prescribe DiHAs. This reluctance to prescribe DiHAs is substantially aggravated by the lack of financial incentives. Through the end of 2022, physicians were reimbursed two euros for the first prescription of DiHA to a patient (55). Since early 2023, physicians have not received any extra compensation for prescribing DiHAs, which is covered by a basic flat fee per quarter for treating a patient insured via the SHI system (14). Consequently, physicians request a higher reimbursement for prescribing DiHAs (54). Overall, only 12% of the GPs believe that their interests have been sufficiently accounted for in the Digital Healthcare Act (54).

Patients, on the other hand, often lack knowledge about digital therapy options (56), which is a prerequisite for accessing DiHAs via the route of self-prescription.

### 3.3. Usage intensity and adherence

#### 3.3.1. Rationale

Adherence to digital therapies is defined as the degree to which a patient follows the program as is was designed (57). In the relevant literature, adherence is often measured as usage intensity in terms of the number of logins or completed modules and has been shown to determine outcomes (58). Thus, if DiHAs are available, prescribed but not used consistently as indicated, their positive impact on the health of the population will be limited. From an SHI perspective, a poor digital therapy adherence constitutes a financial risk because a therapy that has been paid for will not fully benefit patients and result in a waste of SHI funds.

Patient adherence is a particularly important aspect when assessing DiHAs from a population health perspective, as their programs typically require patient collaboration [for example digital CBT (59)] and behavioral changes [for example weight management (60)]. Engaging in health-oriented behaviors and implementing lifestyle changes takes effort and requires motivation (61). Therefore, the risk of digital therapies not being effective owing to a lack of patient adherence is high.

#### 3.3.2. Status quo

Currently, there is no comprehensive assessment of patients' adherence with DiHAs as the market is evolving constantly. A few studies have evaluated patient adherence in specific disease areas. For example, Labinsky et al. (62) found that 54% of patients with rheumatic diseases who were prescribed a DiHA downloaded the app. Of those who downloaded the app, approximately 95% used it at least once, but only 25% completed the entire program. Lack of time and commitment have been reported as the main reasons for nonuse. In a survey conducted by the SHI fund Techniker Krankenkasse, approximately one third of DiHA users were rather dissatisfied with their DiGA with lack of motivation and adherence reported among the reasons (1).

#### 3.3.3. Preliminary conclusions

While there is no comprehensive assessment of patients' adherence with DiHAs, studies of patients' adherence to digital therapies in general report low adherence rates (58). It can thus be expected that patients' adherence to DiHAs also deviates from how their programs have been designed. To determine if and to what extent the reimbursement of DiHAs improves public health, real-world data are needed to investigate how patients interact with apps and how potential behavioral changes translate into health outcomes (63). Currently, the financial risk for SHI funds stemming from low digital therapy adherence is not mitigated.

### 3.4. Patient benefit

#### 3.4.1. Rationale

For the DiHA policy to have a positive impact on the health of the population and on the efficacy of healthcare services, they must benefit patients, either by offering a medical benefit or through an improvement of procedures and structures of healthcare delivery (procedural and structural benefits). Thus, patient benefit is a prerequisite for the policy to be effective.

To foster innovation and expedite market access, the current regulation carries the potential risk that during the period of provisional listing, some DiHAs may be utilized and reimbursed without verifiable evidence of patient benefit. Additionally, there is a risk that DiHAs may secure permanent reimbursement based on studies that could be susceptible to biases [see (28, 29)].

#### 3.4.2. Status quo

Of the 54 DiHAs approved since 2020, six DiHAs (presented in Table 2) have been removed from the directory as efficacy could not be demonstrated during the relevant timeframe of 12 to 24 months. This represents approximately 11% of all approved DiHAs. However, if this number is set in proportion to the number of DiHAs that have been listed but are currently not in their trial period (as they either have the status of permanent listing or were delisted), it becomes evident that 6 out of 27 DiHAs (22%) have not been able to prove a patient benefit. The share of ineffective DiHAs is even higher if only those DiHAs are considered that ended their trial period and were initially approved with provisional listing only (i.e., three DiHAs that obtained a permanent listing from the beginning are not taken into account): 25% of all formerly provisionally listed DiHAs were delisted later.

**Table 2:** Overview of DiHAs delisted from the directory.

Disease area	Name of DiHA (indication)	Duration of trial period	90-day usage cost (in EUR)	Activations (as of 9/2022)	Approx. SHI expenditures (as of 9/2022)
Neurology	M-sense (migraine)	16 months	219.98/10.00	12,000	EUR 1.7 million
Mental health	Selfapy (panic)	17 months	540.00	1,000	EUR 0.5 million
Cardiovascular	Rehappy (stroke)	21 months	299.00/499.00	Not reported individually, part of “others” totaling 1,000	[Total cost not available, maximum based on published numbers: EUR 0.5 million]
Endocrine	Esysta (diabetes)	15 months	249.86		
Oncology	CANKADO PRO-React Onco	24 months	499.80/399.84		
Oncology	Mika (cancer)	12 months	419.00		
DiHAs removed from the directory (6 DiHAs) as a percentage of total DiHAs • Ever listed (54 DiHAs): 11% • Currently not in trial period (27 DiHAs): 22% • With former status of provisional listing (24 DiHAs): 25%					

Table 2 provides an overview of the estimated financial damages to the SHI system. We estimated expenditures by multiplying the average 90-day usage price of a DiHA by the number of activation codes deployed as of September 2022. For the delisted DiHA M-sense (Newsenselab, Germany) against migraine, we showed the actual financial damage reported by the GKV-SV. The amount is lower than the number of activations multiplied by the price since the manufacturer was obliged to partially refund the SHI funds after having requested a removal from the directory—a step the manufacturer took after it had become evident that the approval studies would not demonstrate a patient benefit vs. a placebo. After a dispute between the manufacturer and the GKV-SV, the arbitration court ruled that Newsenselab would have to refund the cost of activated DiHAs during the extension of the provisional approval period of approximately 4 months except for a settlement amount of EUR 10 per activated app. For the DiHAs Cankado PRO-React Onco (CANKADO GmbH, Germany), Mika (Fosanis GmbH, Germany), Esysta (Emperra GmbH E-Health Technologies, Germany), and Rehappy (Rehappy GmbH, Germany), individual activation numbers were not reported, but totaled less than 1,000. Thus, we estimate that the total SHI expenditure on delisted DiHAs, as of September 2022, was approximately EUR 2.7 million or less. With total DiHA expenditures during the same time period equaling EUR 55.5 million, this corresponds to approximately 5%.

Regarding the type of patient benefit of DiHAs, 52 out of 54 DiHAs were approved upon an expected or proven medical benefit. Two DiHAs, namely Cankado PRO-React Onco (CANKADO GmbH, Germany) which has been removed from the directory, and ProHerz (ProCarement GmbH, Germany) solely claim patient relevant structural and procedural benefits.

#### 3.4.3. Preliminary conclusions

The data collected from the DiHA directory show that the risk of reimbursing ineffective DiHAs has materialized. To date, one in four DiHAs that had obtained provisional approval was not able to sufficiently prove a patient benefit under the facilitated approval scheme and low evidence requirements described earlier. Such delistings reduce the trust that healthcare practitioners and patients have in DiHAs, which has a negative impact on prescription rates.

From a financial perspective, the damage to the SHI system thus far is limited. The cost of DiHAs in general and the cost of delisted DiHAs in particular constitute only a minor fraction of the total 2022 SHI benefit expenditures of EUR 274.2 billion, of which EUR 10.4 billion are expenditures for medical devices (64). Hence, expenditures for DiHAs amount to approximately 0.01% of total expenditures.

However, financial damage thus far has been limited primarily because of low prescription numbers. There is yet a lack of mechanisms that limit the financial risks from ineffective DiHAs for the SHI system. The fact that the GKV-SV can seek recourse from manufacturers as in the case of the DiHA M-sense does not fully mitigate this risk because it only applies to the extension of the provisional period. Furthermore, as a as in the case of M-sense, manufacturers may need to file for bankruptcy which further limits the reimbursement amount that can be recovered. Thus, the current policy indirectly shifts part of the R&D risk of DiHAs from manufacturers to the SHI system.

### 3.5. Pricing of DiHAs and SHI resource allocation

#### 3.5.1. Rationale

Pricing is central for an effective resource allocation within a healthcare system; overpricing of proven and effective DiHAs may entail a suboptimal allocation of SHI resources. Contrary to other countries, establishing a monetary value for a universal outcome measure has proven difficult in Germany, primarily because of ethical concerns (8), but services and benefits covered by the German SHI must be “adequate, appropriate and efficient” (35).

For our assessment of this aspect of the policy, we consider the new price after month 13 negotiated between the manufacturer and the GKV-SV or set by the arbitration court as a proxy of an appropriate price, i.e., a price deemed appropriate by the public health system. Based on this, a price decline is an indicator of initial overpricing, and a constant price or price increase is as indication of appropriate pricing.

Furthermore, we consider the number of newly approved DiHAs to be a proxy of research R&D activity in the industry. The number of manufacturers offering DiHAs is a proxy of the attractiveness of the DiHA market for private companies.

#### 3.5.2. Status-quo

Table 3 provides an overview of the price development of permanently listed DiHAs. It also shows the respective group price ceilings that were introduced in October 2022 and April 2023 as well as the duration of the provisional period.

**Table 3:** Price development of DiHAs with permanent listing (for 90 days in EUR).

Name of DiHA by disease area	Initial price	Current price	Price change	Duration of provisional period
Mental health (price ceiling: 599.40)				
▪ Deprexis (GAIA AG)	297.50	210.00	−29%	12 months
▪ HelloBetter depression/ diabetes (GET.ON Institut für online Gesundheitstraining GmbH)	599.00	222.99	−63%	12 months
▪ HelloBetter panic (GET.ON)	599.00	230,00	−62%	0 months
▪ HelloBetter stress/burnout (GET.ON)	599.00	235,00	−61%	12 months
▪ Invirto (Sympatient GmbH)	428.40	620,00	+45%	24 months
▪ Mindable (Mindable Health GmbH)	576.00	576.00	+0%	24 months
▪ Nichtraucher Helden (Sanero Medical GmbH)	239.00	329.00	+38%	24 months
▪ Selfapy (anxiety) (Selfapy GmbH)	540.00	228.50	−58%	15 months
▪ Selfapy (depression) (Selfapy GmbH)	540.00	217.18	−60%	16 months
▪ Somnio (memetor DE GmbH)	464.00	224.99	−52%	12 months
▪ Velibra (GAIA AG)	476.00	230.00	−52%	12 months
▪ Vorvida (GAIA AG)	476.00	192.01	−60%	12 months
Musculoskeletal (price ceiling: 321.30)				
▪ Kaia back pain (kaia health software GmbH)	489.39	489.39	0%	0 months
▪ Vivira (Vivira Health Lab GmbH)	239.96	211.72	−12%	16 months
Neurology (price ceiling: 802.80)				
▪ Elevida (GAIA AG)	743.75	243.00	−67%	12 months
Endocrinology (price ceiling: 513.90)				
▪ Oviva Direkt (Oviva AG)	345.00	411.30	+19%	24 months
▪ Zanadio (aidhere GmbH)	499.80	218.00	−56%	22 months
Otolaryngology (price ceiling: 225.90)				
▪ Kalmeda (mynoise GmbH)	116.97	189.00	+62%	15 months
Urogenital (price ceiling: 670.50)				
▪ Kranus Edera (Kranus Health GmbH)	552.01	656.88	+19%	15 months
Other (no price ceiling)				
▪ HelloBetter Vaginismus (GET.ON)	599.00	235.00	−61%	0 months
▪ HelloBetter chronic pain (GET.ON)	599.00	599.00	+0%	19 months

The prices of permanently listed DiHAs declined substantially across most disease areas. More than half of all permanently listed DiHAs experienced price declines of at least 50%. Within the largest group, mental health, post negotiation prices are substantially below the price ceiling of EUR 599.40. Only a few DiHAs were able to maintain or even increase their prices, including NichtraucherHelden (Sanero Medical GmbH, Germany) for smoking sensation and Invirto (Sympatient GmbH, Germany), which uses virtual reality for anxiety treatment. Two more DiHAs displayed a price increase compared with their original prices; however, those DiHAs increased their prices after listing and later decreased them during negotiations.

In light of these price declines, it is interesting to observe market access activities by manufacturers.

**Figure 3:**
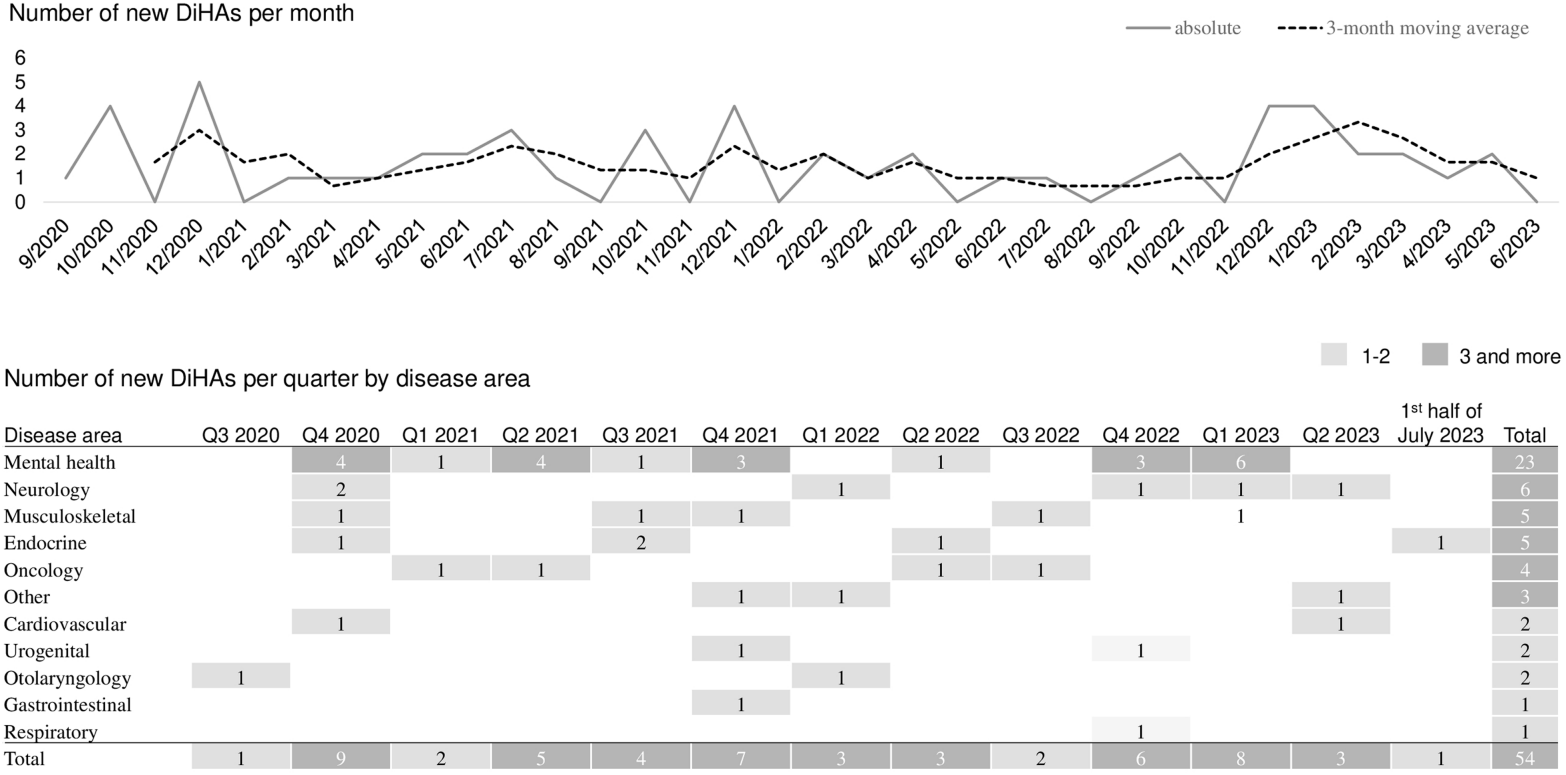
Number of newly approved DiHAs in the market.

Figure 3 shows the monthly number of newly approved DiHAs and the corresponding 3-month moving average. It also displays a quarterly breakdown of the number of newly approved DiHA by disease area. Since September 2020, the number of newly approved DiHAs has remained relatively constant with a monthly average of 1.6 and a standard deviation of 1.4 DiHAs. Figure 3 also illustrates that for all displayed disease areas except respiratory, at least one new DiHA was approved by the end of 2021 and more DiHAs were approved thereafter, except for gastrointestinal where only one DiHA has been approved thus far.

Table 4 provides an overview of all manufacturers in the order of their market entry, i.e., the month of their first DiHA approval. To date, 36 companies have entered the DiHA market, of them 9 in 2020, 12 in 2021, 10 in 2022 and 5 so far in 2023. Thirteen manufacturers offer at least one permanently listed DiHA, 18 offer one or more provisionally listed DiHAs only, and 5 manufacturers no longer have an actively listed DiHA in the directory. Overall, the market is fragmented with 86% of manufacturers offering only one DiHA and another 6% of manufacturers offering two DiHAs. Only three manufacturers have brought six or seven DiHAs to market: GAIA AG, Selfapy GmbH and GET.ON Institut für Gesundheitstrainings GmbH. Those three manufacturers focus primarily on mental health and related conditions and chronic pain (CP). Since 2022, GAIA AG has expanded in oncology (breast cancer) and MS.

**Table 4:** Overview of DiHA manufacturers by time of market entry (month of listing of first DiHA in directory).

Name of manufacturer	Number of DiHAs and respective indications by approval status		
	Permanent	Provisional	Delisted
Mynoise GmbH (9/20)	1: tinnitus	–	–
GAIA AG (10/20)	4: alcoholism, anxiety, depression, MS fatigue	3: borderline, breast cancer, MS	–
Memetor DE GmbH (10/20)	1: insomnia	–	–
Vivira Health Lab GmbH (10/20)	1: back, knee and hip pain	–	–
Aidhere GmbH (10/20)^a^	1: obesity	–	–
Sympatient GmbH (12/20)	1: anxiety	–	–
Newsenselab GmbH (12/20)^b^	–	–	1: migraine
Selfapy GmbH (12/20)	2: anxiety, depression	3: binge eating, bulimia, CP	1: panic
Rehappy GmbH (12/20)^c^	–	–	1: stroke
Fosanis GmbH (3/21)	–	–	1: cancer
Mindable Health GmbH (4/21)	1: anxiety	–	–
CANKADO GmbH (5/21)	-	–	1: breast cancer
Oviva AG (7/21)	1: obesity	–	
Sanero Medical GmbH (7/21)	1: tobacco use	–	
Emperra GmbH E-Health Techn. (7/21)	–	–	1: diabetes
Mawendo GmbH (8/21)	–	1: disease of the patella	–
PrehApp GmbH (10/21)	–	1: kneecap disease	–
IVPNetworks GmbH (10/21)	–	2: anxiety, depression	–
GET.ON GmbH (10.21)	5: CP, depression w/ diabetes, panic, burnout, vaginismus	1: insomnia	–
Kranus Health GmbH (10/21)	1: impotence	–	–
HiDoc Technologies GmbH (12/21)	–	1: irritable bowel syndrome	–
Limedix GmbH (2/22)	–	1: aphasia	–
Sonormed GmbH (3/22)	–	1: tinnitus	–
Vitadio s.r.o., Czech Republic (4/22)	–	1: diabetes type 2	–
PINK gegen Brustkrebs GmbH (6/22)	–	1: breast cancer	–
Kineto Tech Rehab SRL, Romania (9/22)	–	1: osteoarthritis of the knee	–
Endo Health GmbH (10/22)	–	1: endometriosis	–
Perfood GmbH (10/22)	–	1: migraine	–
Elona Health GmbH (12/22)	–	1: depression	–
Kaia health software GmbH (12/22)	1: back pain	1: COPD	–
SOFY GmbH, Austria (12/22)	–	1: depression	–
Smoke Free 23 GmbH (1/23)	–	1: tobacco use	–
Ipso Healthcare GmbH (2/23)	–	1: depression	–
Synaptikon GmbH (5/23)	–	1: mild neuro-cognitive disorder	–
ProCarement GmbH (5/23)	–	1: heart failure	–
Vision2B GmbH (7/23)	–	1: diabetes type 2	–

All manufacturers based in Germany unless stated differently.

^a^ Filed for bankruptcy in May 2023.

^b^ Filed for bankruptcy in July 2022.

^c^ Filed for bankruptcy in September 2022.

To date, three out of 36 DiHA manufacturers have filed bankruptcy. Newsenselab GmbH and Rehappy GmbH after their provisional DiGAs were removed from the directory, aidhere GmbH after the arbitration court cut the reimbursement price by 56% and the manufacturer had to pay back the SHI funds an amount of EUR 10.4 million.

#### 3.5.3. Preliminary conclusions

Overall, these numbers suggest that initial DiGA prices are deemed too high by the GKV-SV and/or the arbitration court. As mentioned, excessive pricing might lead to a suboptimal allocation of resources. This might deter SHI funds from actively informing insured members about digital therapy options as an alternative or complement to traditional therapies.

In the life sciences sector, price levels for healthcare products are commonly justified with the need to recover upfront R&D expenditures (65, 66). Consequently, low reimbursement prices may make business cases unviable, leading to lower investment levels in the sector and fewer R&D efforts.

Currently, there is insufficient data to prove or disprove to what extent the price levels of DiHAs and the observed price cuts after 12 months encourage or discourage R&D efforts. There has not been a systematic study taking into account other factors such as lead times, that might influence the level of innovation activities. However, the development of approval numbers shown in Figure 3 does not suggest a slowdown of development activities in the industry following price cuts. In contrast, both, the absolute approval numbers and their 3-month moving average display a peak in Q4 2022 and Q1 2023 which is largely driven by new mental health DiHAs—a disease area, for which a range of DiHAs already exists and where price cuts had been particularly large.

Additionally, the constant number of new market entrants displayed in Table 4 suggests that the market—despite the observed price reductions—continues to be attractive. To date, one out of 13 manufacturers with permanently listed DiHAs has filed for bankruptcy.

Apparently, Aidhere, the manufacturer of the most DiHA with the highest prescription numbers zanadio against adiposities, was not able to realize scale economies. The company, founded in 2019, grew its headcount rapidly to 150 employees (67). The bankruptcy may not be a necessary result of the price cuts, but a result of management decisions to invest heavily in the development of a multimodal therapy program and corporate support functions (68). It illustrates the trade-off that digital health start-ups face in terms of maintaining a lean structure while having enough expert staff to synchronize product development, scientific studies and the approval process.

## 4. Policy options

In the previous section, we argued that the current DiHA policy in Germany has only partially achieved its goals of fostering healthcare innovation and broad-scale access to it in line with the regulator's task to protect and improve the health of the population. Furthermore, the legislation entails financial risks to the public healthcare system resulting from a suboptimal allocation of public funds.

In the current health political debate, different measures to improve the regulation have been suggested or can be derived from the empirical findings presented in the previous section. Based on this, we first discuss relevant measures and highlight aspects that require future research. Second, we present an integrated directional proposal on how to balance the regulators' interest in fostering innovation in digital health as well as fast, broad-scale access to it and the need for the SHI system to ensure that benefits are adequate, appropriate and efficient.

### 4.1. Individual measures to improve the policy

#### 4.1.1. Directing healthcare innovation

Considering the already large and growing number of DiHAs available in Germany, particularly for mental health, it will be more important to foster innovation in a more targeted way. As the SHI system currently indirectly cross-subsidizes manufacturers' R&D efforts by reimbursing DiHAs without proven benefit, there should be more room for the SHI system to steer those funds toward those indications where innovation might entail the greatest benefit for the population, i.e., those disease areas that are currently underserved and where DiHAs are likely to benefit patients. In the subsequent section on interest alignment between stakeholders, we will discuss an approach on how to achieve this.

#### 4.1.2. Improving patient access

Several measures to increase prescription rates by healthcare professionals can be derived from the studies discussed in 3.2. First, a higher compensation for prescribing DiHAs might increase prescription rates but would put more financial strain on the healthcare system and does not appear to be desired by the SHI funds or by the regulator. Second, an integration of DiHAs into evidence-based treatment guidelines might improve adoption. While the relevant societies are responsible for creating these guidelines, the Ministry of Health is planning to support this process by drafting exemplary digitally supported care processes as part of its digital transformation strategy (69). Third, it is important to educate physicians about DiHAs and to ensure that information on their efficacy is easily accessible and comparable. This comprises a revision of the format in which information within the DiHA directory is presented. In particular, the efficacy of different DiHAs to treat specific conditions should be clearly compared in tabular format. To create transparency on the methodology of evidence creation, a simple, intuitive score should be added to the table which indicates the underlying level and quality of scientific evidence. This score should be derived from existing hierarchical systems of classifying evidence [see, for example (70), for an overview].

Another avenue to improve the health of the population is by increasing the self-prescription volume of proven DiHAs. For some disease areas such as urogenital conditions, more than a third of all DiHAs are accessed via the self-prescription route (16). Increasing self-prescription rates is also in line with the finding that patients' perceived self-efficacy mediates the effect of therapeutic interventions (71). In section 4.2, we will discuss how an increase in self-prescription rates may be achieved.

#### 4.1.3. Adherence to digital therapy programs

As adherence is an important prerequisite for a DiHA to have a positive effect on patient health, the quality of patient engagement should be reflected in the amount reimbursed by the manufacturer. Real-world usage statistics should inform price negotiations or SHI funds might negotiate performance-based prices—for example, in the form of yearly bonuses—if manufacturers achieve specific engagement targets. This would constitute an important incentive for manufacturers to design DiHAs in such a way that they activate patients in real-life settings, for example by following the best practices of persuasive system design and gamification (72). Thus, an important research area is how to design a pricing system that considers usage intensity, including the definition of suitable parameters to measure engagement, how to protect patient privacy, and how to ensure the accuracy and integrity of the data submitted.

#### 4.1.4. Pricing

Similar to considering patient adherence in reimbursement amounts, value-based pricing mechanisms for DiHAs may improve resource allocation in the public healthcare system. There are several taxonomies for the value-based pricing of medicines in general (73) and digital therapeutics in particular (74). For example, Powell and Torous suggested calculating the value of a digital health app based on the country-specific monetary value of the QALY, estimated effect size of the health app, engagement of the patient, and duration of the app's impact (75), with QALY (i.e., quality-adjusted life year) being the academic standard for measuring how effectively a treatment lengthens and/or improves patients' lives (76).

Contrary to other countries, establishing a monetary value for a universal outcome measure has proven difficult in Germany, primarily because of ethical concerns (8). Building on the proposal by the German Advisory Council on the Assessment of Developments in Healthcare to evaluate the appropriateness of DiHA compared to the cost-effectiveness of existing healthcare services covered by the SHI (41), Gensorowsky et al. recently suggested an approach for deriving the appropriate cost of DiHAs (8). Their approach required for each DiHA to first determine an established therapy in the same indication area eligible for reimbursement within the SHI system. For example, cognitive behavioral therapy can be personally delivered for the treatment of depression. Then, the SHI's willingness to pay for a specific improvement in health is calculated by dividing the cost of the existing therapy by the average effect achieved by this therapy option. In the example above, this could be the cost of 15 CBT sessions (approximately EUR 1,500) divided by the average reduction in depressive symptoms (Cohen's d of approximately 0.56). Applying this cost-effectiveness ratio to the average health improvement of a particular DiHA yields an appropriate price that reflects the SHI's willingness to pay to treat a condition. For a DiHA that improves depressive symptoms by a Cohen's d of 0.25, this would be 1,500 EUR/0.56 × 0.25 = EUR 669.64.

While the approach presented by Gensorowsky et al. is a tangible step toward value-based pricing of DiHAs in Germany, there are aspects the framework does not address. For example, it does not consider the possibility of a minimum effect size required for a patient to experience any noticeable positive impact, leading to the question of whether a DiHA with negligible absolute effect should be covered or how to determine a minimum effectiveness threshold. Furthermore, their cost-effectiveness framework does not consider any patient benefits outside of medical benefits. For example, the use of DiHA might have the same health benefits as pharmacological therapy, but with fewer side effects or entail procedural and structural benefits only. These considerations should be considered in future versions of the initial framework.

The value-based framework also assumes that the level of scientific evidence is the same across DiHAs. However, we have seen that the level of scientific evidence and quality of the approval studies conducted varies greatly. Therefore, similar to the suggested evidence score, the regulator might systematically discount the calculated discount prices if they are derived from lower levels of evidence or lower quality studies. In an iterative approach, manufacturers might obtain a higher net reimbursement price upon presenting more robust studies.

From a practical perspective, calculating cost-effective prices is a complex task, as comparable therapies need to be agreed upon, and for each therapy cost-effectiveness measures need to be defined. For manufacturers, this value-based pricing approach entails additional complexity and uncertainty. Especially for manufacturers seeking to develop DiHAs for new disease areas, there is uncertainty as to which therapies will be defined as reference therapies and which metrics will be used to measure effectiveness. Therefore, guidelines are required to define the criteria that should be used to select such measures and comparable therapies. Moreover, research is needed to determine whether such a pricing approach would inhibit certain innovations. For example, there may be indications where pharmacological therapy is inexpensive but has strong side effects; therefore, digital therapy alternatives would be desirable. Finally, research is required to evaluate and detail how value-based prices should reflect the level and quality of underlying scientific evidence.

Both value-based pricing and adherence-based price adjustments require extensive data collected in studies as well as data collected in real-world settings.

#### 4.1.5. Patient benefit

While the possibility of obtaining provisional listing is under high scrutiny especially by the SHI funds, we argue for a modification of its economics such that the policy still maintains its guiding principle of incentivizing innovation and enabling fast, broad-scale access to DiHAs.

A strong advantage of the provisional period is that it allows manufacturers not only to conduct studies, but to also to collect real-world usage data that may be considered in the pricing of DiHAs (see above). Without provisional listing, especially the latter would be challenging for innovative small and medium sized companies in the digital health industry. As such, the provisional period is an enabler of more value-oriented pricing.

Providing a capped reimbursement budget for a DiHA during the provisional listing period is an option for limiting financial risks while encouraging innovation. Such a budget would allow manufacturers to (partially) finance their studies and collect real-world data while placing a clear limit on the financial downsides of SHI funds. If SHI funds obtain more autonomy to define the details of such budgets, this might change the dynamics of the collaboration between manufacturers and payors and put SHI funds, including their regional and national associations, in a position in which they take responsibility and an interest in the development and proliferation of new DiHAs. This idea is further explored in the following subsection.

### 4.2. Integrated framework toward interest alignment between stakeholders

Rather than continuing the antagonistic relationship between SHI funds and manufacturers of DiHAs and their respective associations, aligning their interests would allow the achievement of policy goals more effectively while limiting financial risks for the public healthcare system. SHI funds have a vested interest in improving the care of their insured members. Manufacturers are interested in developing viable digital therapeutic solutions that improve the health of patients. SHI representatives continuously emphasize that proven DiHAs are a suitable means to improve population health; scrutiny is focused on the economic aspects of the relationship between manufacturers and public payers (16). The introduction of value- and usage-based pricing mechanisms for DiHAs with proven patient benefits would be a means to stop economically motivated quarrels between DiHA manufacturers and SHI organizations. Such policies would substantially reduce the financial risks of DiHAs for public payers compared with other therapies.

**Figure 4:**
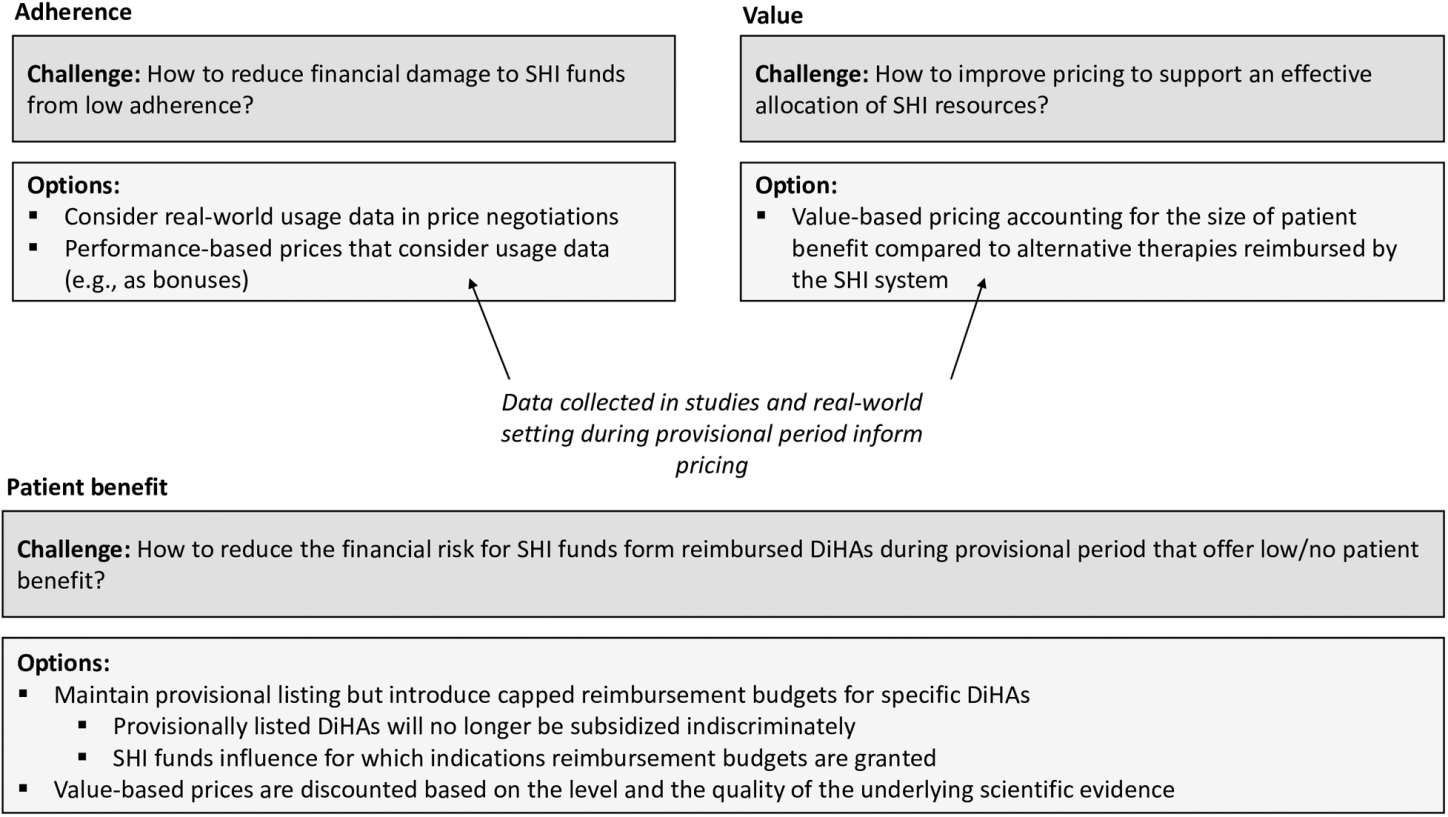
Integrated framework of policy options to align interests and reduce financial risks for the SHI system.

In addition, a modified reimbursement policy during the provisional period may provide opportunities and incentives for SHI funds and their national association to actively steer the development and utilization of DiHAs to the benefit of their insured members. In principle, SHI funds are in an excellent position to steer the development of new DiHAs because they have information about the conditions their insured members are suffering from and treatments. Based on their data, SHI funds can identify care gaps and indications for which new DiHAs would be particularly useful. SHI funds are also in an excellent position to increase the utilization of DiHAs in the population as they can educate their insured members about DiHAs and how to access them if needed. SHI funds launching information campaigns among their insured members on DiHAs have the potential to substantially increase their self-prescription rates. Figure 4 displays the integrated framework to align the interests of stakeholders.

A new reimbursement policy regarding the provisional period that aligns the interests of all stakeholders would need to comprise two aspects: (1) Risk mitigation: Instead of exposing SHI funds to unlimited financial risk, the regulator might introduce a maximum reimbursement budget for DiHAs entering the provisional period. Consequently, manufacturers would still be financially supported in their R&D efforts, but the financial risk for SHI funds would be calculable. (2) Budget authority: Currently, SHI organizations must indirectly bear a part of the R&D costs without being in a position to influence which R&D efforts are funded and with which budget. We suggest enabling the GKV-SV to influence the size of reimbursement budgets for DiHAs with provisional listings in a structured manner. This can be achieved through a scoring scheme developed by GKV-SV, which determines the size of the reimbursement budget during the trial period. This scoring scheme considers factors such as:

- Size of the patient population suffering from the indication that a new DiHA addresses.
- Perceived gap in traditional care, for example, due to capacity constraints in stationary or ambulatory care or side effects of medication.
- Cost of traditional care.
- The number of existing DiHAs addressing this indication (the more DiHAs are already available, the lower the need for new solutions, and thus, the lower the score).
- Preliminary assessment of how well a digital therapy would be suited to improving patient health in the respective disease areas.
- Other factors include the complexity of technology development (e.g., if elements of virtual reality are required higher budgets might be needed).

By enabling SHI funds to influence the size of a reimbursement budget for new DiHAs, policymakers can influence the two challenges we described in our framework that relate to the achievement of policy goals: how to ensure that the right products are developed (i.e., where there is a need for innovation) and how to increase (self-) prescription rates. Therefore, along with value- and usage-based pricing mechanisms, the general concept of reimbursement budgets for DiHAs with provisional listings is an important avenue for future research.

## 5. Recommendations

Defining the details of a value- and usage-based pricing mechanism as well as the size, scoring logic, and process of reimbursement budgets for DiHAs with provisional listing, are important steps for the German public health system to extract more value from DiHAs while protecting public funds from the ineffective allocation of resources. However, these steps, including intensified research efforts, will require at least 1–3 years, while the financial risks are immediate. Especially regarding DiHAs without proven patient benefits, there is a risk of abuse, with companies launching questionable apps for 12 months just to delist them before having to submit final evidence.

Therefore, we recommend implementing protective mechanisms in the short term. This would require the regulator to enable SHI funds to obtain downside protection, which can be achieved through reimbursement budget caps for DiHAs without proven patient benefits (or other mechanisms such as reinsurance).

Regarding the medium-term implementation of value- and usage-based pricing mechanisms, it is advisable to use a staggered approach. Value- and usage-based pricing frameworks should be defined sequentially, beginning with the most important disease areas with sufficient lead time so manufacturers can prepare for it. The process should provide room for feedback from the market, particularly manufacturers, to ensure that an increase in pricing complexity does not outweigh the incentives for innovation that stem from the reimbursement of DiHAs in the public health insurance system.

## 6. Conclusions

This policy review showed that the reimbursement of digital health applications via the public health insurance system in Germany has been accompanied by a strong increase in digital therapy options for patients. The regulator acknowledges the impact of the regulation on the pace with which innovation is made available to patients on a broad-scale as the definition of DiHAs will be extended to higher risk class IIb medical devices such as monitoring devices of vital parameters such as oxygen saturation or heart rate as well as blood glucose (41, 69).

However, prescription rates are very low, which constitutes a major hurdle for the policy to have a noticeable effect on population health. Furthermore, we demonstrate that a number of inherent financial risks remain in the public system that are not fully mitigated. In Germany, these financial risks are the main reason that public payers are still skeptical toward DiHAs and are often seen launching campaigns against their overpricing (77, 78) rather than informing healthcare practitioners and patients about their benefits. This situation is regrettable, as public payers may play a pivotal role in informing the relevant population about the existence and benefits of DiHAs. Therefore, our study focused on the mechanisms to achieve better interest alignment between manufacturers and public payers. In an ideal scenario, SHI funds and their regional or national associations would support manufacturers in developing the appropriate solutions to benefit their insured members, not only by providing funds and identifying disease areas that would benefit from innovation. SHI funds or their associations may even create units or accelerator programs that actively support young companies through expertise and coaching, for example, regarding study design or providing input for product design.

Our recommendations for introducing value- and usage-based pricing mechanisms, scores that reflect the level and the quality of scientific evidence as well as reimbursement budgets for DiHAs with provisional approval are directional. Further research is required to determine the specificities.

Other countries may turn to Germany as a case study to determine which aspects of the policy would apply beneficially to their national systems and where refinements are needed. While our recommendations are relevant to most countries with a developed public health insurance system, local regulations, the roles of different stakeholders and the maturity of the digital health innovation ecosystem will influence to what extent they are applicable.
